# Sleep disordered breathing in mucopolysaccharidosis I: a multivariate analysis of patient, therapeutic and metabolic correlators modifying long term clinical outcome

**DOI:** 10.1186/s13023-015-0255-4

**Published:** 2015-04-10

**Authors:** Abhijit Ricky Pal, Eveline J Langereis, Muhammad A Saif, Jean Mercer, Heather J Church, Karen L Tylee, Robert F Wynn, Frits A Wijburg, Simon A Jones, Iain A Bruce, Brian W Bigger

**Affiliations:** Department of Paediatric Otolaryngology, Royal Manchester Children’s Hospital, Manchester, UK; Stem Cell & Neurotherapies, Faculty of Medical and Human Sciences, University of Manchester, Manchester, UK; Department of Paediatric Metabolic Diseases, Emma Children’s Hospital, Academic Medical Centre, Amsterdam, The Netherlands; Department of Haematology/BMT, Royal Manchester Children’s Hospital, Manchester, UK; Willink Biochemical Genetics Unit, Manchester Centre for Genomic Medicine, St. Mary’s Hospital, Manchester, UK

**Keywords:** Lysosomal storage disease, Mucopolysaccharidosis type I, Hurler syndrome, Haematopoietic stem cell transplant, Enzyme replacement therapy, Sleep-disordered breathing, Obstructive sleep apnoea syndrome, Inhibitory antibodies

## Abstract

**Background:**

The lysosomal storage disorder, mucopolysaccharidosis I (MPS I), commonly manifests with upper airway obstruction and sleep disordered breathing (SDB). The success of current therapies, including haematopoietic stem cell transplantation (HSCT) and enzyme replacement therapy (ERT) may be influenced by a number of factors and monitored using biomarkers of metabolic correction. We describe the pattern of SDB seen in the largest MPS I cohort described to date and determine therapies and biomarkers influencing the severity of long-term airway disease.

**Methods:**

Therapeutic, clinical and biomarker data, including longitudinal outcome parameters from 150 sleep oximetry studies were collected in 61 MPS I (44 Hurler, 17 attenuated) patients between 6 months pre to 16 years post-treatment (median follow-up 22 months). The presence and functional nature of an immune response to ERT was determined using ELISA and a cellular uptake inhibition assay. Multivariate analysis was performed to determine significant correlators of airway disease.

**Results:**

The incidence of SDB in our cohort is 68%, while 16% require therapeutic intervention for airway obstruction. A greater rate of progression (73%) and requirement for intervention is seen amongst ERT patients in contrast to HSCT treated individuals (24%). Multivariate analysis identifies poorer metabolic clearance, as measured by a rise in the biomarker urinary dermatan sulphate: chondroitin sulphate (DS:CS) ratio, as a significant correlator of increased presence and severity of SDB in MPS I patients (*p* = 0.0017, 0.008). Amongst transplanted Hurler patients, delivered enzyme (leukocyte iduronidase) at one year is significantly raised in those without SDB (*p* = 0.004). Cellular uptake inhibitory antibodies in ERT treated patients correlate with reduced substrate clearance and occurrence of severe SDB (*p* = 0.001).

**Conclusion:**

We have identified biochemical and therapeutic factors modifying airway disease across the phenotypic spectrum in MPS I. Interventions maximising substrate reduction correlate with improved long-term SDB, while inhibitory antibodies impact on biochemical and clinical outcomes. Monitoring and tolerisation strategies should be re-evaluated to improve detection and minimise the inhibitory antibody response to ERT in MPS I and other lysosomal storage diseases. Future studies should consider the use of sleep disordered breathing as an objective parameter of clinical and metabolic improvement.

## Background

Mucopolysaccharidosis I (MPS I) is a rare multisystem lysosomal storage disorder inherited in an autosomal recessive manner with an incidence between 1.07 and 3.8 per 100,000 [1,2]. A deficiency in the enzyme α_1_- L- iduronidase (IDUA) results in pathological accumulation of the glycosaminoglycans (GAGs) dermatan sulfate (DS) and heparan sulfate (HS) [3]. A spectrum of severity in clinical presentation has been recognized with 50% to 80% of patients classified into a severe phenotype (MPS I Hurler) with the remainder distributed into attenuated groups (MPS I Hurler-Scheie and Scheie). Current therapeutic strategies include haematopoietic stem cell transplantation (HSCT) for the Hurler phenotype and enzyme replacement therapy (ERT) in attenuated cases [4,5].

Life threatening involvement of the respiratory system is a well-recognised feature of MPS. A common manifestation of upper airway disease in MPS I is with sleep disordered breathing (SDB) and obstructive sleep apnoea syndrome (OSAS) [6,7]. Sleep disordered breathing describes a spectrum of symptoms and signs characterized by an abnormal respiratory and ventilatory pattern during sleep, encompassing snoring, upper airways resistance syndrome and OSAS. These occur as a consequence of increased upper airway resistance due to the multilevel skeletal, oral, adenotonsillar, laryngeal and tracheal involvement seen in MPS I [8-10]. OSAS is characterized by recurrent episodes of partial or complete airway obstruction during sleep with oxygen desaturations [11]. Multichannel polysomnography is able to measure the severity of these episodes based on an apnoea-hypopnoea index, while resultant desaturations may be measured by overnight transcutaneous oximetry. Failure to recognise, and treat, moderate to severe SDB has behavioural and physiological consequences, including failure to thrive, neurocognitive and developmental delay and, in severe cases, cardiorespiratory sequellae [12,13].

The existing literature describing the incidence of OSAS in MPS is based on cross sectional analysis of small cohorts, primarily performed prior to initiation of treatment [6,7,14,15]. Short-term longitudinal data is available from the early Phase III ERT trials, assessing the role of a single therapeutic intervention on OSAS [16-18]. We describe sleep oximetry data in the largest MPS I cohort to date identifying demographic and biochemical factors, including the impact of inhibitory antibodies, that affect the presence and severity of sleep disordered breathing following treatment with either ERT or HSCT.

## Methods

MPS I patients, treated at the Royal Manchester Children’s Hospital, Manchester, UK and the Academic Medical Centre, Amsterdam, The Netherlands between 2003–2013, were identified from existing patient databases. A study group of 61 patients in whom long-term follow-up was available at the two centres were included in the study. Patients were recruited following consent to donate blood, which is routinely sought from all MPS patients in Manchester with appropriate ethical approval (REC No. 08/H1010/63, North West Research Ethics Committee; Central Manchester University Hospital NHS Foundation Trust Research & Development Committee). The clinical notes of patients were retrospectively reviewed for overnight sleep oximetry studies, therapeutic and biochemical data.

### Clinical investigations and biomarker endpoints

The primary endpoint measured was the presence, progression and severity of sleep disordered breathing (SDB), based on overnight sleep oximetry data. Secondary endpoints included leukocyte IDUA enzyme activity one year post HSCT, the urine dermatan sulphate: chondroitin sulphate (DS:CS) ratio biomarker and requirement for therapeutic airway intervention following commencement of treatment for MPS.

#### Sleep disordered breathing endpoints based on overnight sleep oximetry

Studies were performed during un-sedated natural sleep, using sleep oximetry equipment (Pulsox 300i, Konica Minolta) measuring transcutaneous oxygen saturation and pulse rate with a finger monitor. Sleep studies were performed routinely as part of annual assessment guidelines, prior to general anaesthesia or if clinically indicated by symptoms [4,19].

The presence and severity of significant SDB was determined by oxygen desaturation index 4% (ODI4%), percentage of time spent below 90% oxygen saturation (SpO2) and the median SpO2. Based on guidelines from the Working Party on Sleep Physiology and Respiratory Control Disorders in Childhood, Royal College of Paediatrics and Child Health (2009) [20] the criteria of abnormality in nocturnal oximetry recordings are falls of more than 4% below baseline oxygen saturation and transient desaturations below 90%. Based on normative data, a normal oximetry recording should have a median SpO2 level greater than 95% and no more than 4 desaturations of 4% or greater per hour (ODI4%: 5–10 moderate, >10 severe SDB) [21,22].

**Figure 1:**
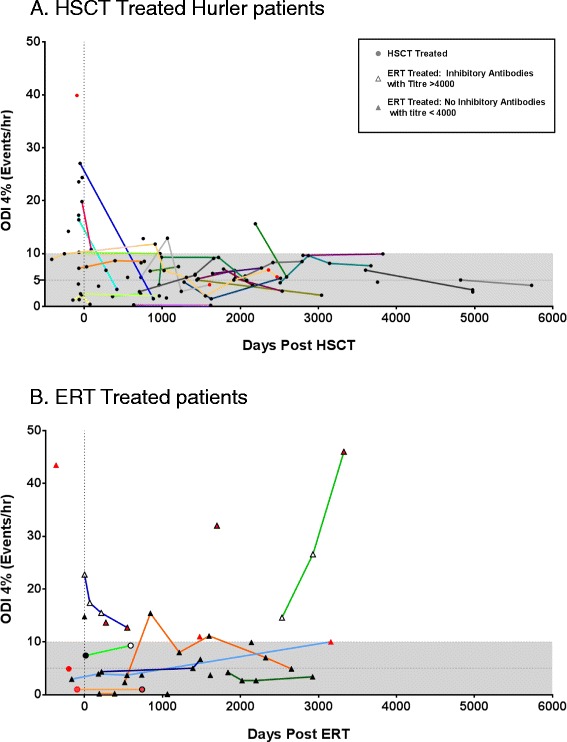
**Longitudinal trends in sleep disordered breathing (SDB) following treatment in MPS I patients treated with A. haematopoietic stem cell transplant (HSCT) or B. enzyme replacement therapy (ERT).** Oxygen desaturation index 4% (ODI4%) data from sleep studies for each individual patient is plotted against duration since initiation of treatment, with solid lines connecting trends per patient. The shaded area represents an ODI4% of less than 10, representing the cut-off for severe SDB. Red legend identifies patients who require therapeutic intervention to the airway following the identified sleep study. **(A)**. Data for 41 patients is presented. Hurler patients (circles) treated with HSCT show improvement in the severity of SDB compared to pre-treatment. Over the duration of follow up the majority of patients (76%) improve or remain stable. A reducing proportion of patients suffer severe SDB, with only one patient demonstrating severe SDB in the period after 3 years post HSCT. 3 patients require therapeutic intervention post-HSCT. **(B)**. Data in 17 ERT treated attenuated patients (triangles) and 3 Hurler patients (circles) is presented. Amongst attenuated patients with longitudinal data, 73% are seen to progress, but rarely to severe SDB However, a cohort of attenuated patients developing inhibitory antibodies (open triangles) continue to suffer with severe SDB. 7 patients require therapeutic intervention (surgery or CPAP) while on ERT.

Binary endpoints were determined for the presence of significant SDB (ODI 4% > 5 events per hour and median saturation <95%) and for progression (deterioration in ODI4% of greater than 1 event/hour, and 1% or greater drop in median SpO2 in studies more than 1 year apart). Studies performed in patients following a therapeutic surgical airway intervention, such as adenotonsillectomy, laryngeal microsurgery or continuous positive airways pressure (CPAP), while on treatment for MPS were excluded from the analysis and are indicated in Figure 1.

#### Urinary glycosaminoglycan and enzyme assay for α-L-iduronidase activity

Measurement of DS:CS ratio biomarker was performed by the Willink Biochemical Genetics Laboratory, St. Mary’s Hospital, Manchester, UK as part of routine clinical monitoring of disease progression from urine, by two-dimensional electrophoresis on cellulose-acetate sheets [23]. GAGs were separated into discrete spots and located after staining with Alcian blue and washing with 5% Acetic Acid. Semi-quantitative measurement of the area of two-dimensional electrophoresis spots of extracted GAG were made under white light using the UVP Gel Photography system and LabWorks software (Anachem, Luton, UK) [24,25]. This biomarker provides a measure of the degree of pathological GAG substrate accumulation in comparison to non-pathologically stored chondroitin sulphate.

The activity of α-L-iduronidase (IDUA) was measured on leucocytes isolated from peripheral blood based on catabolism of the artificial fluorescent substrate 4-methylumbelliferyl-α-L-iduronide (Glycosynth, Warrington, UK) as described previously [26]. Enzyme activity was expressed as μmol/g total protein/hour (normal reference range 10–50, heterozygote range 5–25).

#### Antibody detection and cellular uptake inhibition assay

Patient serum antibody titres towards ERT and cellular uptake inhibition were measured using two assays as described previously [27]. Briefly, the antibody detection assay used an immunoglobulin G enzyme linked immunosorbent assay (ELISA) to detect and quantify patient serum antibodies against recombinant IDUA enzyme (Aldurazyme, Genzyme, Framingham, USA, 10 μg/ml). Antibody titre for each patient sample was based on two-fold serial dilutions of serum starting from a 1:8 to a 1:262,144 dilution. Control serum, from patients without MPS, was used to define a baseline. The cut off values for antibody titre for the ELISA were determined using the following formula for each dilution.

$$ \begin{array}{l} Cut\  of f\  value = \left[ Mean\  absorbance\  of\  patient\  sample\ \hbox{--}\ 1SD\right]\ \\ {}\kern3.96em \hbox{--}\ \left[ Mean\  absorbance\  of\  normal\  serum + 2SD\right]\end{array} $$

A value above zero indicates a positive result. The titre above the last negative value (last positive dilution) is reported as the positive titre for that patient sample. The cellular uptake inhibition assay quantifies the level of catalytically active recombinant enzyme taken up into cultured enzyme naive MPS I patient fibroblasts. The ability of enzyme to catalyse a fluorescent artificial substrate (4MU-α-L-iduronide) is measured. Cellular uptake of enzyme proceeds via the Mannose-6-Phosphate (M-6-P) ligand receptor uptake pathway. Rather than testing only for inhibition of catalytic activity, neutralisation of enzyme uptake via the M-6-P pathway is also assessed. Cultured cells are incubated with enzyme and antibodies from patient sera. Prior to assessing fluorescence, extracellular enzyme that has not been successfully internalised is washed. Therefore, following cell lysis the substrate assay only quantified intracellular enzyme. The protein content of each assay well was corrected for using the Pierce Bicinchoninic acid (BCA) assay (Thermoscientific) according to the manufacturer’s instructions.

The % inhibition of the catalytic activity was measured as follows:

$$ \%\  Inhibition = 100 - 100 \times \frac{Enzyme\  activity\  of\  lysate\  incubated\  with\  patient\  serum}{Enzyme\  activity\  of\  lysate\  incubated\  with\  normal\  serum\  control} $$

### Statistical analysis

Descriptive statistics were calculated for demographics, sleep oximetry and biochemical data. Significant associations between patient, biomarker and treatment-related variables and primary endpoints for all patients, HSCT treated Hurler and ERT treated attenuated subgroups were identified using multivariate stepwise regression modelling using the minimum corrected Akaike information criterion (AICc), followed by logistic regression for binomial outcomes and standard least squares for continuous outcomes. The relationship between these variables is individually presented. For continuous outcomes, to ensure equal weighting, the mean value for sleep oximetry data and timepoints were calculated. Significance was assumed where 95% confidence intervals did not include 1 (*p-values <0.05*). Possible confounding variables including age at start of treatment and duration of follow-up were included in the model. JMP v11.0 software (SAS) was used for this analysis. For correlation plots, linear regression lines of best fit were drawn and correlation coefficients were calculated with Pearson’s r and Spearman’s rho for normally and non-normally distributed data sets respectively. *T*-test and Mann–Whitney tests were used to identify differences between the baseline characteristics of subgroups.

## Results

### Study demographics

Clinical data for 61 patients, with a median age of 6.8 years at the time of final assessment, were collected (median duration of follow-up 22 months, range 1–60 months). Forty-four patients were classified as severe Hurler based on genotype and enzyme studies at diagnosis (median age at follow-up 4.7 years, range 10 months – 16 years, median follow-up duration 637 days), while 17 were diagnosed as the attenuated phenotype (median 9.7 years, range 5.2-35 years, median follow-up duration 595 days). Forty-one of 44 Hurler were treated with HSCT with a median age at transplantation of 14 months old, while 3 Hurler patients were treated with ERT alone due to delayed diagnosis and advanced disease at initiation of treatment (mean 8.4 years, range 6.2-12 years). All 17 attenuated patients were treated with ERT, with a median age at start of treatment of 60 months. Subject demographic and treatment characteristics are presented in Table 1.

**Table 1:** **Baseline patient and treatment characteristics**

**Patient characteristics:**		**N**	**%**	**Median**	**Range**
**All MPS I**		61			
	Gender (male/female)	38/23	62/38		
	Phenotype (Hurler/Attenuated)	44/17	69/31		
	Age @ Start of treatment (mths)			18	3-364
	Age @ final assessment (mths)			82	0.3-420
**HSCT treated Hurler patient characteristics**					
	Gender (male/female)	30/14			
	Age @ Start of treatment (mths)			14	3-30
	Age @ final assessment (mths)			66	11-203
	**Interventions**				
	Therapeutic airway intervention post-HSCT	3	7%	Adenotonsillar surgery: 2 pts (1 had revision). Longterm O_2_: 1	
	**Treatments and metabolic characteristics**				
	No HSCT (1/2/3)	32/8/1	78/20/2		
	Source (CB/BM/PBSC/Unknown)	17/15/7/2	41/37/17/5		
	Donor (Related/MUD)	15/26	37/63		
	IDUA @ 1 year post HSCT	36		30.0	6.3-87.0
	Heterozygote Donors	12		19.7	6.7-49.4
	Matched Unrelated Donors	24		34.6	17.7-87.0
	Pre HSCT DS:CS ratio	20		1.6	0.7-3.4
	DS:CS ratio @ 1 year	32		0.5	0.2-0.8
**ERT treated Hurler Patients**					
	Gender (male/female)	2/1			
	Age @ Start of treatment (mths)			85	74-144
	Age @ final assessment (mths)			123	98-148
**ERT Treated attenuated patients**					
	Gender (male/female)	9/8			
	Age @ Start of treatment (mths)			60	24-364
	Age @ final assessment (mths)			131	72-420
	**Interventions**				
	Therapeutic airway intervention on ERT	7	41%	CPAP: 5 pts	
				Adenotonsillar Surgery: 2 pts (1 had revision)	
	**Metabolic characteristics**				
	Pre ERT DS:CS ratio	5		1.9	1.1-2.7
	DS:CS ratio @ 1 year	13		0.8	0.4-2.2

All characteristics concern the last HSCT. BM indicates bone marrow; CB, cord blood; IDUA, alpha-L-iduronidase enzyme level; mths, months; MUD, matched unrelated donor; N, number; PBSC, peripheral blood stem cells.

### Primary endpoint: sleep disordered breathing

Based on analysis of parameters from 127 post-treatment sleep oximetry studies, significant SDB was present in 68% (36/53, ODI4% > 5/hr) of our cohort. Table 2 summarises the descriptive statistics for sleep oximetry results including for phenotype and therapy subgroups.

**Table 2:** **Sleep oximetry characteristics**

**All patients**		**N**	**%**	**Missing**	**Median**	**Range**
	No of Studies	150			2 per pt	1.0-9.0
	Duration since treatment start (years)				3.3	0.1-15.7
	Duration of final assessment since treatment start (years)		8 pts pre-treatment studies only		4.4	0.1-15.7
	Patients with sleep disordered breathing (present/absent)	36/17	68/32	8 (SDB present in 6/8 with only pre-treatment studies)		
	Progression of sleep disordered breathing (yes/no)	15/22	41/59	24		
	ODI 4% Post treatment				6.1	0.1-46.0
	Median oxygen saturation Post treatment				95.4	83.9-98.1
	% Time < 90% Oxygen saturation Post treatment				2.2	0.0-30.8
**HSCT treated Hurler patient**						
	No of Studies	106				
	Time post treatment start (years)				3.1	0.1-14.9
	Sleep disordered breathing post HSCT present/absent	24/11	69/31	6		
	Progression of SDB in patients with multiple studies yes/no	6/19	24/76	16		
	No of pre-treatment studies	19			In 14 patients	
	Pre-treatment ODI 4%				9.5	1.3-39.9
	Pre-treatment Median oxygen saturation				96.2	81.6-98.5
	Pre-treatment % Time < 90% Oxygen saturation				4.1	0-14.2
	No. of post treatment studies	87			In 35 patients	
	ODI 4% Post treatment				5.8	0.2-19.8
	Median oxygen saturation Post treatment				95.4	87.4-99.6
	% Time < 90% Oxygen saturation Post treatment				1.75	0-30.8
**ERT treated Attenuated Patients**						
	No of Studies	44				
	Time post treatment start (years)				3.5	0.7-8.0
	Sleep disordered breathing (SDB) present	11/6	65/35			
	Progression of SDB yes/no	8/3	73/27	7		
	No of pre-treatment studies	4			In 3 patients	
	Pre-treatment ODI 4%				5.7	0.2-14.8
	No of post treatment studies	40			In 17 patients	
	ODI 4% Post treatment				6.9	0.1-46
	Median oxygen saturation Post treatment				95.1	83.9-98.1
	% Time < 90% Oxygen saturation Post treatment				3.2	0-17.25

N indicates number; SDB, Sleep disordered breathing.

ODI 4%, oxygen desaturation index 4%.

Amongst post-transplant Hurler patients, SDB was present (ODI > 5%) in 69% but seen to progress in only 24%. In those with both pre and post HSCT studies (n = 8) a significant improvement in the severity of SDB was seen after treatment (median ODI4%; pre HSCT: 9.5/hr vs. post HSCT 4.1/hr; p = 0.02). Longitudinal analysis of HSCT treated Hurler patients demonstrate that 13% (4/30) patients had evidence of severe OSA (ODI4% > 10) after assessment at 1 and 2 years post HSCT respectively. Only one individual (5%, 1/21) exhibited severe SDB at follow-up of greater than 3 years post HSCT (Figure 1A).

Following ERT initiation in attenuated patients, SDB was deemed to be present in 65% (ODI > 5%), but seen to progress in 73% (Figure 1B; ERT vs. HSCT, p = 0.013). Thirty-six percent (5/14) of those with follow up over 1 year have severe SDB and this increased to 45% (5/11) with studies after 3 years of ERT.

#### Airway interventions

Sixteen percent (10/61) of the study population required 13 episodes of therapeutic airway intervention following initiation of ERT/HSCT (Table 1), with a later age at start of treatment found to significantly correlate with the need for intervention (p = 0.012). The proportion of attenuated patients treated with ERT necessitating intervention was 41% (7/17). All patients requiring CPAP were from the ERT treated attenuated subgroup (n = 5), while 2 necessitated airway surgery alone. The proportion of HSCT treated Hurler patients undergoing intervention post HSCT was significantly lower at 7% (3/41; ERT vs. HSCT, p = 0.01) composed of one patient requiring long term oxygen therapy and 2 managed with adenotonsillectomy.

#### Secondary endpoints and correlators of sleep disordered breathing

Correlator variables for the outcomes of presence, progression and severity of long term SDB, as measured by sleep oximetry were determined following multivariate analysis (Table 3). Potential confounders, including age at commencement of treatment and duration of follow-up were included in the analysis.

**Table 3:** **Correlators of sleep related clinical outcome based on multivariate analysis**

**Outcome**	**Modifier**	**Patient group**	**Exp (β)**	**Lower 95% CI**	**Upper 95% CI**	**p-value**
**SDB Present**	Phenotype	All (n = 61)				ns
	Treatment					ns
	Age @ start of treatment					ns
	Follow-up duration					ns
	DS:CS ratio @ 1 year		−5.94	−12.39	−1.61	0.0017
**Mean ODI 4%**	Phenotype	All (n = 61)				ns
	Treatment					ns
	Age @ start of treatment					ns
	Follow-up duration					ns
	DS:CS ratio @ 1 year		6.35	1.77	10.95	0.008
**Therapeutic Intervention**	Phenotype	All (n = 61)				ns
	Treatment					ns
	Age @ start of treatment		−0.21	−0.45	−0.03	0.012
	Follow-up duration					ns
	DS:CS ratio @ 1 year					ns
**SDB Present**	Donor Type	HSCT Treated Hurler (n = 41)				ns
	IDUA level @ 1 year		0.08	0.02	0.16	0.004
	Age @ start of treatment					ns
	Follow-up duration					ns
	DS:CS ratio @ 1 year					ns
**ODI 4% at time of antibody status**	DS:CS Ratio	ERT Treated Attenuated (n = 17)				ns
	Inhibitory Antibodies		−8.27	−11.19	−5.36	0.001
	Age @ start of treatment					ns
	Follow-up duration					ns

*p*-values for modifiers based on multivariate analysis using stepwise fit (minimum AICc) followed by standard least squares for continuous outcomes and logistic fit for binomial outcomes. CI indicates confidence interval; ERT, enzyme replacement therapy; HSCT, haemopoietic stem cell transplant; IDUA, alpha-L-iduronidase enzyme level; SDB, Sleep disordered breathing; ODI 4%, oxygen desaturation index 4%. Inhibitory antibodies defined as IgG titre > 4000 and uptake inhibition >30%.

#### DS:CS ratio and metabolic correction

DS: CS ratio was available in 24 patients prior to treatment and post-ERT/HSCT in 47. No significant difference was present between pre-treatment attenuated and severe patients (median DS:CS; 1.9 vs 1.6, p = 0.59), while paired data demonstrated a significant improvement in post-therapy levels following both ERT (median pre and post ERT DS:CS; 1.9 vs. 0.6, n = 5, p = 0.02) and HSCT (median pre and post HSCT DS:CS; 1.6 vs 0.4, n = 19, p < 0.0001).

**Figure 2:**
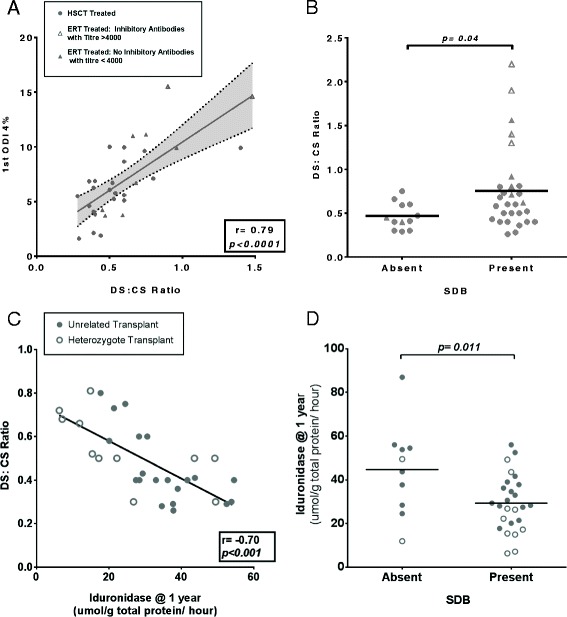
**Metabolic biomarkers of clinical outcome in MPS I.** Substrate reduction, measured by DS:CS ratio, and delivered enzyme activity following HSCT correlate significantly with measures of sleep disordered breathing (SDB) amongst MPS I patients. Bars represent means with p values presented from Mann–Whitney U (Multivariate p values are presented in Table 3). For correlation plots, linear regression lines of best fit were drawn and correlation coefficients were calculated with Pearson’s r, with p values representing significantly non-zero lines of best fit. Hurler patients identified by circle legend and attenuated by triangles. Each individual point represents one patient. **(A)**. The ODI4% from the first post treatment sleep study of each patient correlates strongly to urinary DS: CS ratio performed at an identical time point (sleep study and urine sample collected within 4 weeks). Pearson Correlation r = 0.79 (r^2^ = 0.68), p < 0.0001. **(B)**. Mean urinary DS:CS ratio 1 year after treatment was significantly improved in individuals without SDB (mean 0.47, S.D 0.15), compared to those with SDB (mean 0.75, S.D 0.48, p = 0.04). Amongst ERT treated patients, patients with inhibitors drive worsening SDB. **(C)**. Increasing iduronidase (IDUA) one year following transplant correlates significantly with improved DS:CS ratio at one year (r = −0.70, p < 0.001). **(D)**. Iduronidase enzyme activity one year post transplant is significantly higher when SDB was absent (mean 44.69, S.D 20.8), compared to those where SDB was present (mean 29.33, S.D 12.9, p = 0.011). HSCT, Matched unrelated donor (MUD): closed circles. HSCT, Heterozygote donors: open circles. Attenuated patients on ERT without antibody response: closed triangles: Attenuated ERT patients with inhibitors: open triangles.

Metabolic correction, measured by a fall in the biomarker DS:CS ratio, correlates significantly with clinical regression of airway disease following treatment, as measured by ODI4% at an identical time point (oximetry within 4 weeks of urine sample) (Figure 2A (p < 0.0001)). This association continues to be significant amongst the entire cohort in the multivariate analysis, correcting for age at start of treatment and at assessment, for the presence of SDB (Figure 2B; Table 3: DS:CS @ 1 year and presence (multivariate p = 0.0017) and severity of SDB (p = 0.008)).

#### IDUA enzyme activity post-HSCT

Engrafted leukocyte IDUA enzyme activity was documented at one-year post transplant (Table 1). Stratified by donor type, patients receiving HSCT demonstrated greater enzyme activity (Table 1; median IDUA, heterozygote: 19.7 vs unrelated 34.6, p = 0.01) and greater substrate clearance (median DS:CS ratio; heterozygote 0.40 vs unrelated 0.47, p = 0.04) when receiving grafts from unrelated donors with two copies of the missing IDUA gene as opposed to heterozygote, related donors expressing one gene copy. Greater enzyme levels were seen to confer greater substrate clearance (Figure 2C, *p* < 0.001). IDUA enzyme level at one year post HSCT correlated significantly for the presence of SDB, correcting for age at HSCT and at assessment (p = 0.011, Figure 2D).

#### Immune response to ERT

**Figure 3:**
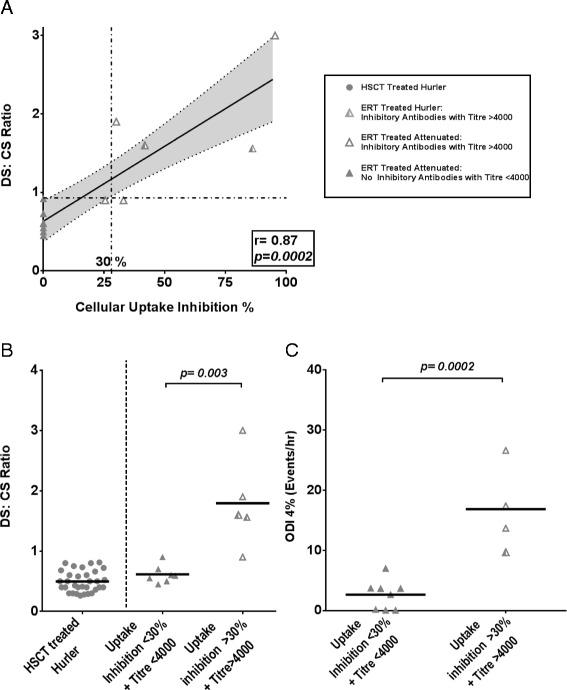
**The role of inhibitory antibodies on metabolic biomarker and sleep disordered breathing (SDB) amongst ERT treated MPS I patients.** Bars represent means with p values presented. For correlation plots, linear regression lines of best fit were drawn and correlation coefficients were calculated with Pearson’s r, with p values representing significantly non-zero lines of best fit. HSCT treated Hurler patients: closed circles. Hurler patients treated with ERT: Half shaded triangles. ERT patients with clinically significant inhibitory antibodies were defined as a cellular uptake inhibition greater than 30% & titres greater than 4000 (Open triangles). ERT treated patients without such an antibody response represented by closed triangles. **(A)**. Correlation plot with linear regression lines of best fit for cellular uptake inhibition and DS:CS ratio. Increasing activity of inhibitory antibodies correlates strongly with poorer substrate clearance (urinary DS:CS ratio performed within 6 months of antibody status) (r^2^ = 0.74, p = 0.002). 30% uptake inhibition is a suggested percentage with a measurable biochemical effect based on intersection of upper confidence intervals at baseline and lower CI. **(B)**. Urinary DS:CS ratio within 6 months of antibody status was significantly improved in individuals without inhibitory antibodies (mean 0.61, S.D 0.14) compared to those with inhibitors (mean 1.8, S.D 0.76). As a reference, ERT attenuated patients without inhibitors have as successful metabolic outcomes as HSCT treated Hurler patients (mean 0.47). **(C)**. SDB as measured by ODI4% within 6 months of antibody status was significantly lower in individuals without inhibitory antibodies (mean 2.03, S.D 1.68) compared to those with inhibitors (mean 16.85, S.D 7.23).

The results from the antibody detection and cellular uptake inhibition assay are described in Table 4. Increasing inhibition of enzyme activity by antibodies correlated significantly with poorer substrate reduction (DS:CS ratio) in ERT patients, Figure 3A (p = 0.0002). Inhibitory antibodies were deemed effectual in patients with both a raised IgG antibody titre above 1/4000 dilution and evidence of cellular uptake inhibition above 30% (based on extrapolation from the upper CI at baseline and its intersection of the lower CI; Figure 3A).

**Table 4:** **ERT related immune response**

**Patient no.**	**Phenotype**	**Age at start of ERT (Yrs)**	**Age at assessment of antibody status**	**Antibody titre**	**Cellular uptake inhibition %**	**Inhibitory antibodies beyond 1 year**
**1**	Hurler	11	13*	262336	86.1	Yes*
**2**	Hurler	7	7	65536	41.8	No
**3**	Hurler	6	7	262144	88.3	Yes
**4**	Hurler-Scheie	5	7*	262336	0	No
**5**	Hurler-Scheie	4	4*	32768	33.0	Yes*
**6**	Hurler-Scheie	5	13*	32768	95.4	Yes*
**7**	Hurler-Scheie	8	15	256	0	No
**8**	Hurler-Scheie	6	13*	512	0	No
**9**	Hurler-Scheie	3	9*	1	n/a	No
**10**	Hurler-Scheie	3	10*	256	0	No
**11**	Hurler-Scheie	7	14*	1	n/a	No
**12**	Hurler-Scheie	12	12	4096	0	No
**13**	Hurler-Scheie	2	7*	1	n/a	No
**14**	Hurler-Scheie	4	4	32768	25.5	No
**15**	Hurler-Scheie	4	7*	256	30.09	No
**16**	Hurler-Scheie	4	7*	32	1	No
**17**	Scheie	30	30	131072	58.10	No
**18**	Scheie	27	28*	262144	30.12	Yes*
**19**	Scheie	15	15	4096	11.6	No
**20**	Scheie	9	10*	2048	31.4	No

*Sleep study and biochemical data available within 6 months of determination of antibody status.

Eleven (52%) individuals were identified as having IgG antibody titres greater than 4000. Of these, 7 (33%) demonstrated uptake inhibition above 30%. All Hurler patients treated with long term ERT (not treated with HSCT, n = 3) in this cohort demonstrated a strong inhibitory antibody response. Patients with titres above 4000, and uptake inhibition above 30% had significantly worse DS:CS ratios (performed within 6 months) than those without inhibitory antibodies, Figure 3B; p = 0.003. Notably, ERT corrected patients without inhibitors had similar DS:CS ratios to Hurler patients receiving HSCT (Figure 3B).

Of the 4 attenuated patients, 3 demonstrated both high titres and uptake inhibition over 1 year after ERT had started and had SDB and biochemical data available within 6 months of antibody status determination (Identified with asterisk, Table 4). These 3 patients began ERT aged 4.5 years, 5.9 years and 26.9 years respectively. All demonstrated evidence of severe SDB (ODI > 10%) on assessment, and have required therapeutic intervention while on ERT (CPAP in 2, Surgery in 1; Figure 1B). Patients with significant levels of inhibitory antibodies all showed significantly elevated SDB over ERT treated patients without inhibitory antibodies, as confirmed by multivariate analysis (Table 4; Figure 3C; open triangle legend).

## Discussion

This is the first paper to present long-term post-treatment outcomes for airway obstruction in MPS I and to correlate objective parameters of clinical airway disease with both metabolic correction and levels of inhibitory antibodies. We see more advanced clinical airway disease in patients with poorer substrate clearance, indicated by raised DS:CS ratio. Hence, factors optimising substrate reduction and engrafted delivered enzyme in HSCT, primarily non-carrier donor selection and full donor chimerism [24,25], will have a beneficial effect on respiratory outcome; whereas, amongst ERT treated individuals, factors compromising metabolic clearance such as inhibitory antibodies to ERT and delayed treatment impair clinical outcome.

These results endorse the ability of sleep oximetry to discriminate patients based on severity of metabolic disease and subsequent correction following treatment, and suggest oximetry could serve as quantitative and clinically relevant primary outcome parameter representative of clearance of GAGs and derived polysaccharides responsible for obstructive airway disease. Recent studies have qualitatively confirmed the utility of signs of upper airway obstruction as a means to differentiate between severe and attenuated phenotypes in MPS [28]. Furthermore, the clear relationship between metabolic correction, delivered enzyme and clinical outcome suggests that in the future, autologous stem cell gene therapy, as a means to maximise disease correction based on supra-normal enzyme levels [29,30], may further minimise the continuing burden of disease in this population.

From our analysis of long term outcome, HSCT appears to provide sustained metabolic correction and subsequent reduction in airway obstruction, with only a small proportion suffering with progressive or severe SDB after 3 years post-transplant. In contrast, a greater proportion of ERT managed attenuated patients are seen to progress, require therapeutic intervention and suffer severe SDB. These findings strengthen the description of other studies that fewer airway related complications are seen following successful HSCT than ERT [31]. However, it must be borne in mind that a formal comparison of clinical outcomes between HSCT and ERT treated groups is necessarily limited in a retrospective study such as this, due to the heterogeneous nature of untreated disease, inequalities in time of assessment and variation in age at onset of symptoms and disease severity at start of treatment. Prospective evaluation would also be hampered by the variation in age at presentation.

One conclusion that may be drawn from our results is that attenuated patients, receiving their first ERT on average 46 months after Hurler patients receive HSCT, are more refractory to treatment as they are older, with more established clinical disease at initiation of treatment. This includes an older cohort diagnosed prior to the era of ERT. Whilst acknowledging the significance of age at start of treatment and follow-up duration in affecting clinical outcome, our multivariate analysis demonstrated DS:CS ratio and inhibitory antibodies to still be significant correlators of SDB taking into account the above confounders.

Further analysis amongst ERT patients, identifies two groups of patients. Almost all of the worsening metabolic (Figure 3B) and SDB effects (Figure 3C), are mediated by a small cohort of ‘non-responders’ with clinically meaningful, high levels of inhibitory antibodies; while the majority, without such a response, demonstrate excellent biochemical and clinical improvement. Allowing for low patient numbers and variation in time points, this effect continues to prove significant following analysis accounting for age at start of treatment and at follow-up. This suggests that both HSCT and ERT would be equally effective at reducing metabolic and airway obstruction if inhibitory antibodies could be eliminated in ERT.

Although recombinant protein replacement therapies in several diseases, particularly Pompe and to a lesser extent haemophilia, have been shown to have a negative clinical response to inhibitory antibody induction [32], this has not been the case in MPS I. 91% of MPS I patients treated with laronidase on the original ERT trial developed IgG or IgM allo-antibodies to recombinant enzyme [16], although no evidence of poor clinical outcome has been demonstrated in MPS until now. The cellular uptake assay to measure inhibitory antibodies, used in conjunction with stratification into subsets with high IgG titres is required to truly evaluate the impact of such antibodies and provides a more meaningful reflection of *in vivo* processes required for effective substrate clearance with ERT compared to an in vitro enzyme catalytic inhibition assay alone [27]. This has recently been clearly correlated with several metabolic biomarkers, including DS:CS ratio [33]. The strong correlation seen between DS:CS ratio and ODI4% reasserts our findings that an allo-immune response that impairs substrate clearance is likely to reduce the clinical efficacy of ERT in MPS and merits further prospective collaborative investigation using a standardized assay in a larger cohort. Thus presence of greater than 30% cellular inhibition, whilst removing patients with clinically ineffectual low IgG titres, delineates between patients with worse SDB from those with improved SDB (Figure 3C).

We acknowledge the limitations of our study, including cohort size, especially amongst the ERT group, and retrospective nature of data collection. Full multichannel polysomnography was not available in a significant proportion of patients, as a result, formal quantification of OSA based on apnoea-hypopnoea index (AHI) was not possible; however, correlation between AHI and ODI in patients undergoing both studies was good and therefore sleep oximetry data was used. Non-invasive oximetry is well tolerated, and we were able to perform studies in almost all patients including those with advanced disease. Data on the use of sleep oximetry for the identification of OSA have suggested that when positive, the results show good correlation with PSG, but a potentially poor predictive value if results were negative [21] [34]. This potential error was minimised given the high incidence of SDB in our cohort and as the majority of patients underwent multiple studies.

## Conclusion

As a chronic disease with poorly defined global clinical outcomes, being able to demonstrate a clear correlation between clinical airway obstruction and metabolic correction is a significant finding. The findings of this study have a number of potential implications for the current management of SDB in MPS I. Firstly, optimising metabolic correction, monitored by biomarker response, can be seen to improve respiratory outcome. We also identify that HSCT in Hurler patients and ERT in attenuated individuals without inhibitory antibodies results in sustained correction of airway disease. However, a cohort of attenuated patients demonstrates advanced disease, which appears to be driven by raising inhibitory antibody responses. The correlation between worsening substrate reduction and SDB to inhibitory antibodies requires further investigation and suggests that monitoring of inhibitory antibodies and investigation of tolerisation regimens to prevent such a response is needed to form part of routine management of ERT treated patients in future. Alternatively, as the management of risk in HSCT improves, it may become feasible as a single treatment modality for both severe and significantly affected attenuated phenotypes of MPS I.
